# Supplementary material for: Bioorthogonal in situ assembly of nanomedicines as drug depots for extracellular drug delivery
Source: Nat Commun. 2022 Apr 19;13:2038. doi: 10.1038/s41467-022-29693-8 (PMC9018704; doi:10.1038/s41467-022-29693-8)
Supplement: Supplementary file 1 — Supplementary Informaton [file 41467_2022_29693_MOESM1_ESM.pdf]

Supplementary Information

Bioorthogonal in situ assembly of nanomedicines as drug depots for extracellular drug delivery

Ziyang Cao[1,2], Dongdong Li[2], Liang Zhao[3], Mengting Liu[2], Pengyue Ma[1], Yingli Luo [3,4], Xianzhu Yang[1,2,3,4*]

Affiliations:

[1] Department of general surgery, Guangzhou First People's Hospital, South China University of Technology, 510006 Guangzhou, P. R. China

[2] School of Biomedical Sciences and Engineering, South China University of Technology, Guangzhou International Campus, 511442 Guangzhou, P. R. China

[3] National Engineering Research Center for Tissue Restoration and Reconstruction, and Key Laboratory of Biomedical Engineering of Guangdong Province, South China University of Technology, 510006 Guangzhou, P. R. China.

[4] Key Laboratory of Biomedical Materials and Engineering of the Ministry of Education, and Innovation Center for Tissue Restoration and Reconstruction, South China University of Technology, 510006 Guangzhou, P. R. China

*Correspondence should be addressed to Prof. X.Z. Yang (yangxz@scut.edu.cn)

Supplementary Fig. 1 The synthetic route of **a** Boc-Cys and **b** Cys-PEG-*b*-PLA polymer.

Supplementary Fig. 2 The synthetic route of CBT-PEG-*b*-PLA polymer.

Supplementary Fig. 3 ^{1}H NMR spectra of Boc-Cys in DMSO-d_6.

Supplementary Fig. 4 ^{1}H NMR spectra of Boc-Cys-PEG-*b*-PLA in DMSO-d_6.

Supplementary Fig. 5 ^{1}H NMR spectra of Cys-PEG-*b*-PLA in DMSO-d_6.

Supplementary Fig. 6 ^{1}H NMR spectra of CBT-PEG-*b*-PLA in DMSO-d_6.

Supplementary Fig. 7 The synthetic route of DA-Cys-PEG-*b*-PLA.

Supplementary Fig. 8 ^{1}H NMR spectra of DA-Cys-PEG-*b*-PLA in DMSO-d_6.

Supplementary Fig. 9 The stability of D-NP, C-NP and the mixed D-NP/C-NP in PBS solution containing 10% FBS.

Supplementary Fig. 10 ^{1}H NMR spectra of crosslinked blank D-NP and C-NP.

Supplementary Fig. 11 The synthetic route and ^{1}H NMR spectra of of SA-Cys-PEG-*b*-PLA.

Supplementary Fig. 12 The stability of S-NP and the mixed S-NP/C-NP in PBS solution containing 10% FBS.

Supplementary Fig. 13 ^{1}H NMR spectra of synthetic hydrophobic platinum prodrug (IV) in DMSO-d_6.

Supplementary Fig. 14 Pharmacokinetics curve in blood and biodistribution of platinum prodrug-loaded S-NP/C-NP or D-NP/C-NP after i.v. administration to BALB/c mice bearing an orthotopic 4T1 tumor.

Supplementary Fig. 15 The biodistribution of Cy5-labeled D-NP/C-NP and S-NP/C-NP in major organ and tumor at 48 h or 96 h i.v. post-injection.

Supplementary Fig. 16 The release behavior of encapsulated BB94 from S-NP$_{BB94}$/C-NP$_{BB94}$ and D-NP$_{BB94}$/C-NP$_{BB94}$.

Supplementary Fig. 17 *In vitro* assembly of D-NP/C-NP decrease the cellular uptake.

Supplementary Fig. 18 Series CLSM images of live 4T1 cells co-incubated with Cy5-labeled D-NP/C-NP or S-NP/C-NP.

Supplementary Fig. 19 The extracellular/intracellular NPs or BB94 quantitative analysis experiment.

Supplementary Fig. 20 The average growth curves of orthotopic 4T1 breast tumor after different treatment in Fig. 4.

Supplementary Fig. 21 Balb/c mice bearing orthotopic 4T1 tumors were treated as indicated in Fig. 4a.

Supplementary Fig. 22 *In vivo* pharmacokinetic profiles of D-NP, S-NP and C-NP.

Supplementary Fig. 23 The release behavior of DOX, NLG919 and BLZ945 from drug-loaded nanoparticles.

Supplementary Fig. 24 The body weight changes of mice in the treatment of Fig. 6b.

Supplementary Fig. 25 Tumor histological analysis with TUNEL and Ki-67 staining after different treatments.

Supplementary Fig. 26 Gating strategy for immune cell analysis by flow cytometry.

Supplementary Fig. 27 Ratio of $CD8^{+}$ T cells to Treg cells in tumor after treated as indicated in Fig. 6b.

Supplementary Fig. 28 Representative flow cytometric analysis images of M1-like macrophages.

Supplementary Fig. 29 Representative flow cytometric analysis images of M2-like macrophages.

Supplementary Fig. 30 Ratio of M1-like macrophages to M2-like macrophagse in tumor after treated as indicated in Fig. 6b.

Supplementary Fig. 31 Representative immunofluorescence images of CD8, CD4, CD206 and CD86 in tumor sections.

Supplementary Fig. 32 Intratumoral Kyn to Trp ratio examined after treated as indicated in Fig. 6b.

Supplementary Fig. 33 The uncropped versions of immunoblot images in Fig. 4d.

Supplementary Figures

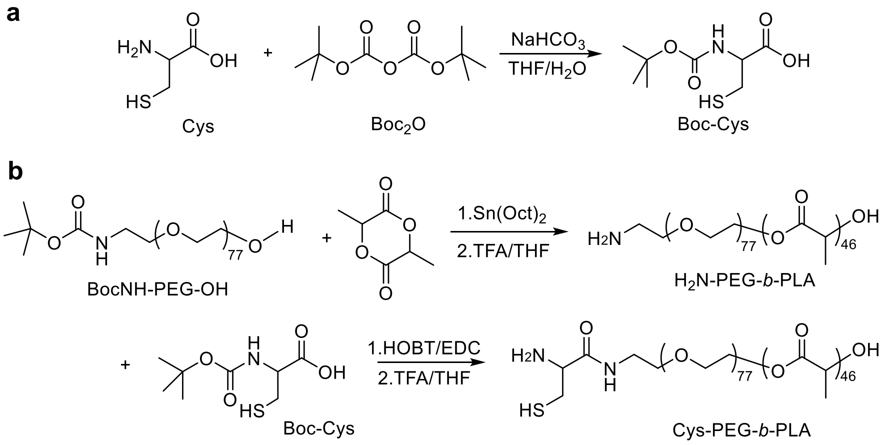

Supplementary Fig. 1 The synthetic route of **a** Boc-Cys and **b** Cys-PEG-*b*-PLA polymer.

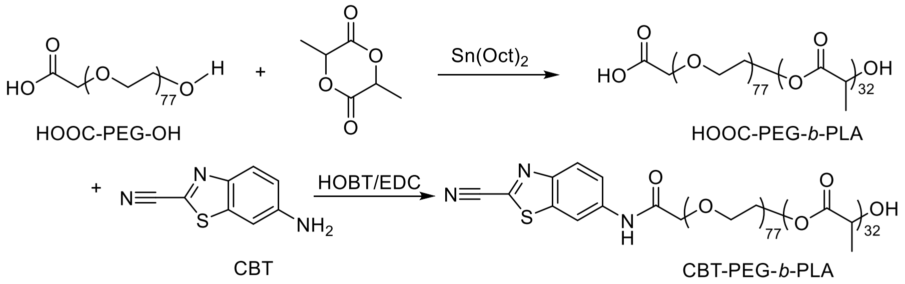

Supplementary Fig. 2 The synthetic route of CBT-PEG-*b*-PLA polymer.

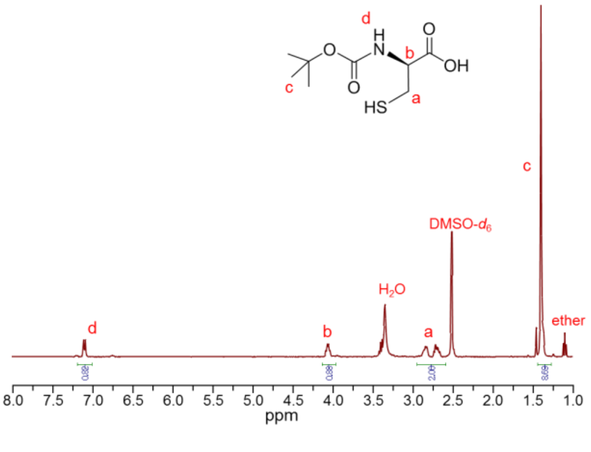

Supplementary Fig. 3 ^{1}H NMR spectra of Boc-Cys in DMSO-d_6 recorded on an

NMR Bruker AVANCE III 400 MHz spectrometer at 25 °C (ppm).

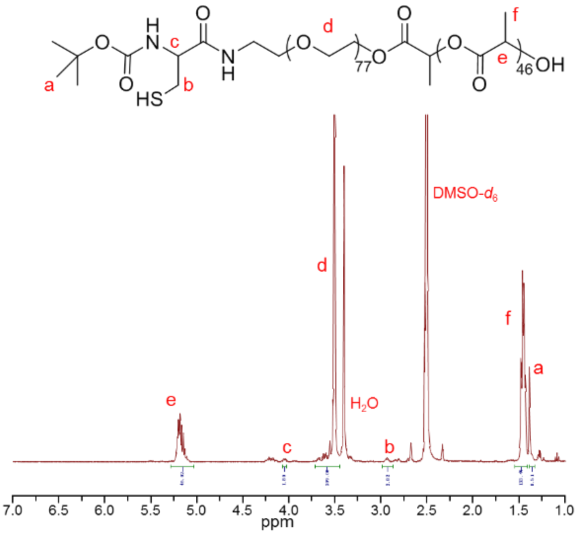

Supplementary Fig. 4 ^{1}H NMR spectra of Boc-Cys-PEG-*b*-PLA in DMSO-d_6 recorded on an NMR Bruker AVANCE III 400 MHz spectrometer at 25 °C (ppm).

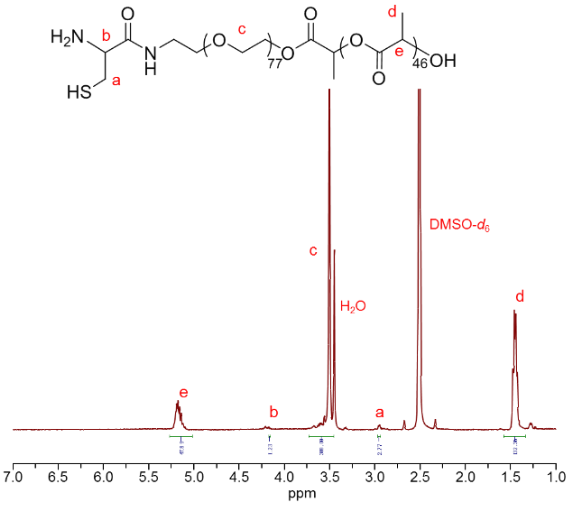

Supplementary Fig. 5 ^{1}H NMR spectra of Cys-PEG-*b*-PLA in DMSO-d_6 recorded on an NMR Bruker AVANCE III 400 MHz spectrometer at 25 °C (ppm).

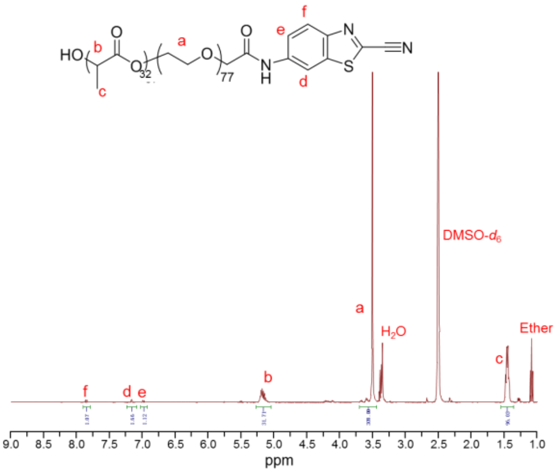

Supplementary Fig. 6 ^1H NMR spectra of CBT-PEG-*b*-PLA in DMSO-d_6 recorded on an NMR Bruker AVANCE III 400 MHz spectrometer at 25 °C (ppm).

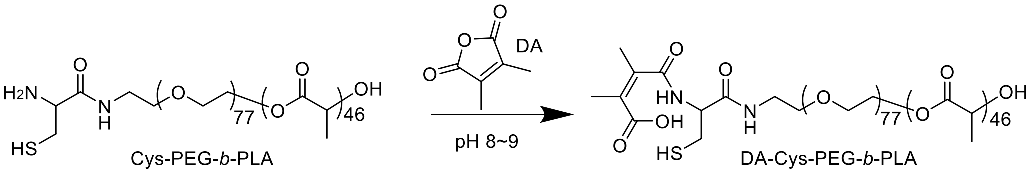

Supplementary Fig. 7 The synthetic route of DA-Cys-PEG-*b*-PLA.

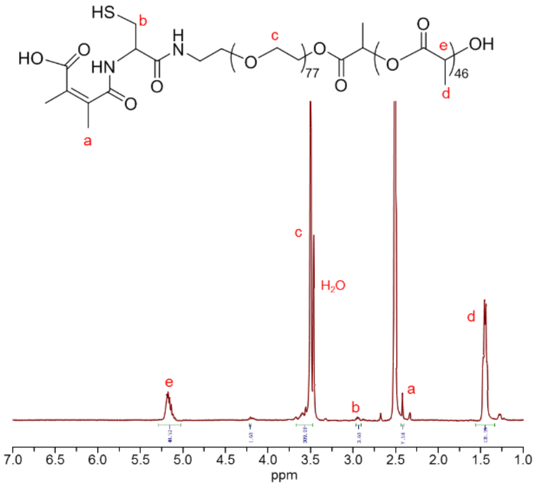

Supplementary Fig. 8 ^1H NMR spectra of DA-Cys-PEG-*b*-PLA in DMSO-d_6 recorded on an NMR Bruker AVANCE III 400 MHz spectrometer at 25 °C (ppm).

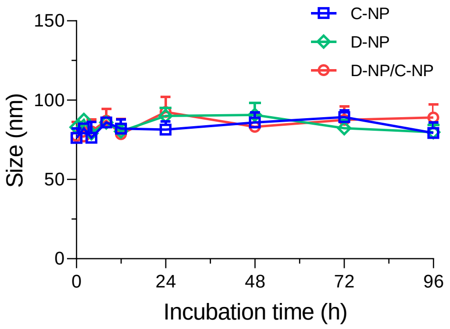

Supplementary Fig. 9 D-NP, C-NP and the mixed D-NP/C-NP were incubated with PBS solution containing 10% FBS for different periods, and the size variation were examined by the Zetasizer Nano ZS Instrument. Data were presented as the mean ± s.d. (n = 3).

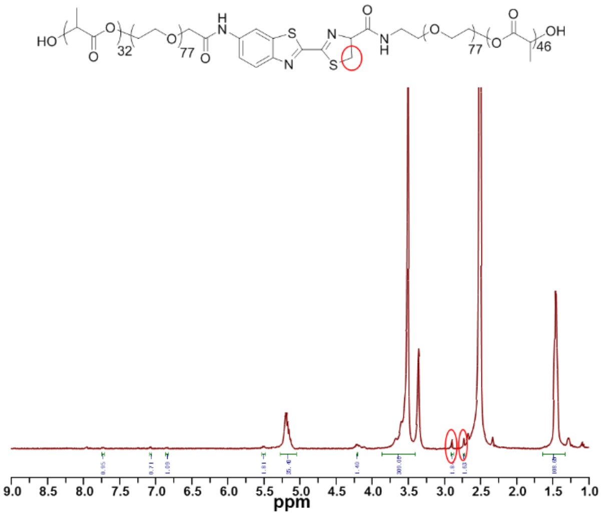

Supplementary Fig. 10 ^{1}H NMR spectra of blank D-NP and C-NP stirred at pH 6.5 for 2 h in DMSO-d_6 recorded on an NMR Bruker AVANCE III 400 MHz spectrometer at 25 °C (ppm).

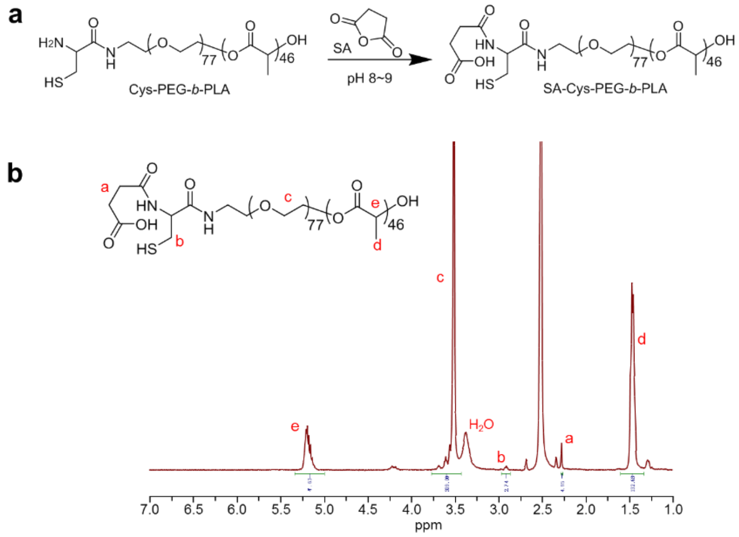

Supplementary Fig. 11 a The synthetic route of SA-Cys-PEG-*b*-PLA and **b** ^{1}H NMR spectra of SA-Cys-PEG-*b*-PLA in DMSO-d_6 recorded on an NMR Bruker AVANCE III 400 MHz spectrometer at 25 °C (ppm).

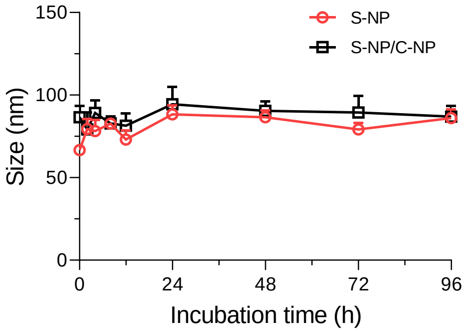

Supplementary Fig. 12 S-NP and the mixed S-NP/C-NP were incubated with PBS solution containing 10% FBS for different periods, and the size variation were examined by the Zetasizer Nano ZS Instrument. Data were presented as the mean ± s.d. (n = 3).

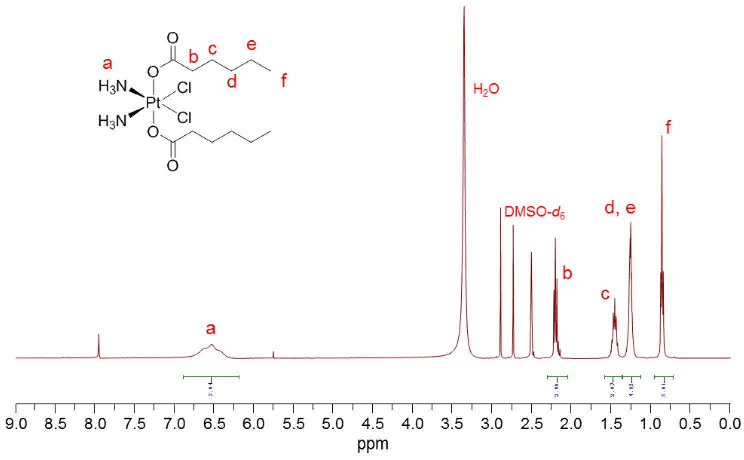

Supplementary Fig. 13 ^{1}H NMR spectra of synthetic hydrophobic platinum prodrug (IV) in DMSO-d_6 recorded on an NMR Bruker AVANCE III 400 MHz spectrometer at 25 °C (ppm).

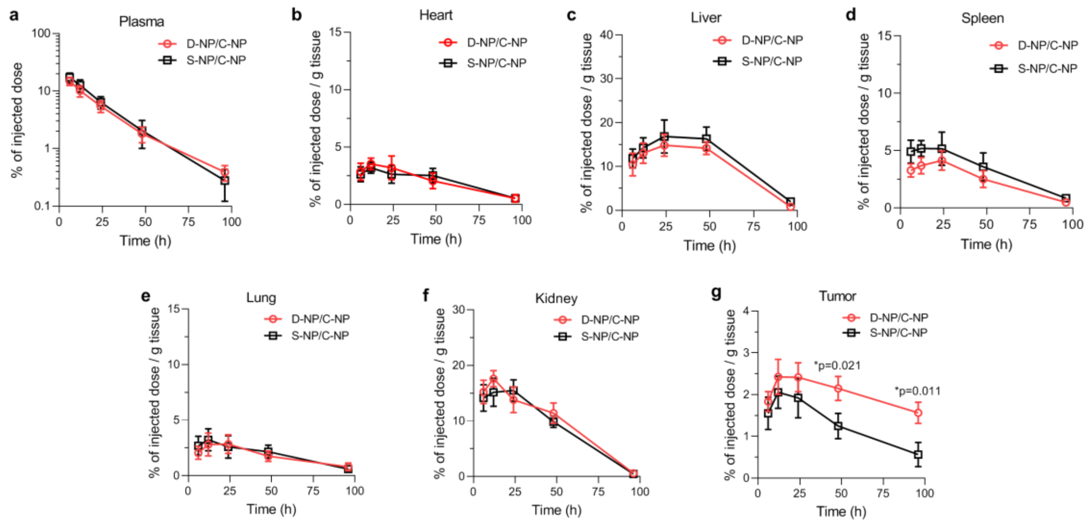

Supplementary Fig. 14 Pharmacokinetics curve in blood and biodistribution of platinum prodrug-loaded S-NP/C-NP or D-NP/C-NP after i.v. administration to BALB/c mice bearing an orthotopic 4T1 tumor. Time course of the contents of platinum in plasma (**a**) and the accumulation of platinum in heart (**b**), liver (**c**), spleen (**d**), lung (**e**), kidney (**f**) and tumor (**g**) after i.v. injection at a platinum dose of 40 μg/mouse at different time points (6 h, 12 h, 24 h, 48h and 96 h). Data were presented as the mean ± s.d. (n = 3). Statistical significance was calculated *via* student's t-test **(two tails). *p< 0.05.**

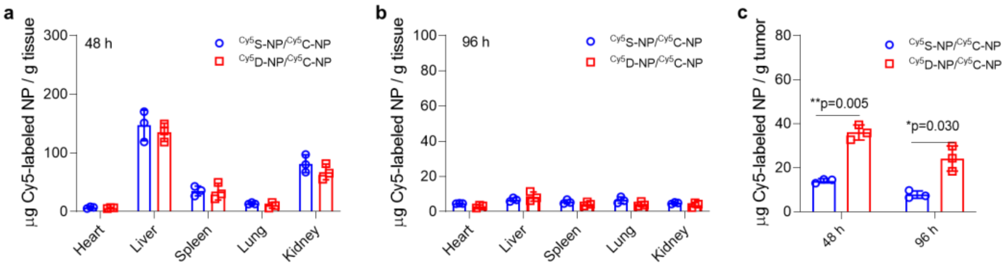

Supplementary Fig. 15 The biodistribution of Cy5-labeled D-NP/C-NP and S-NP/C-NP in major organ (**a, b**) and tumor (**c**) at 48 h or 96 h i.v. post-injection. Data were presented as the mean ± s.d. (n = 3). Statistical significance was calculated *via* student's t-test (two tails). *p< 0.05, **p<0.01.

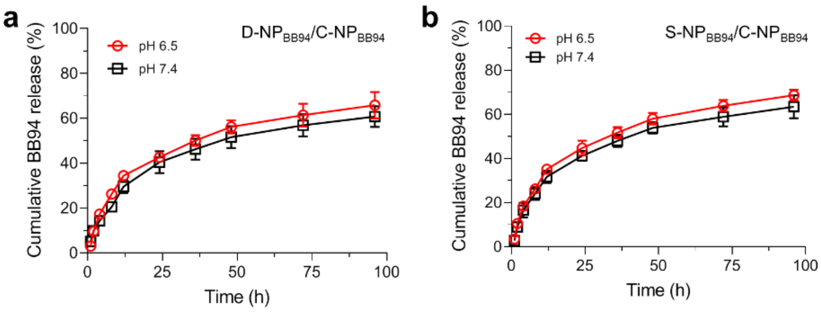

Supplementary Fig. 16 The release behavior of encapsulated BB94 from S-NP$_{BB94}$/C-NP$_{BB94}$ and D-NP$_{BB94}$/C-NP$_{BB94}$ under different pH environment. Data were presented as the mean ± s.d. (n = 3).

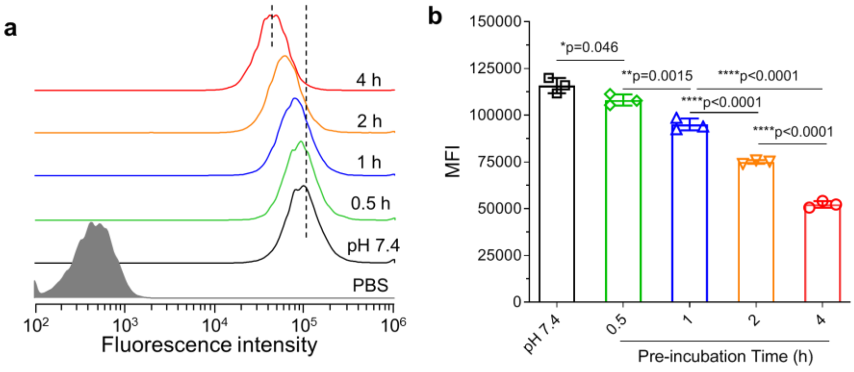

Supplementary Fig. 17 *In vitro* assembly of D-NP/C-NP decrease the cellular uptake. **a** FACS analysis and **b** mean fluorescence intensity (MFI) of 4T1 cells incubated with Cy5-labeled D-NP/C-NP after pre-incubation at pH 6.5 for different times (0.5 h, 1 h,

2 h, 4 h). Data were presented as the mean ± s.d. (n = 3). Statistical significance was calculated via one-way ANOVA with a Tukey post-hoc test. *p<0.05, **p<0.01, ****p< 0.0001.

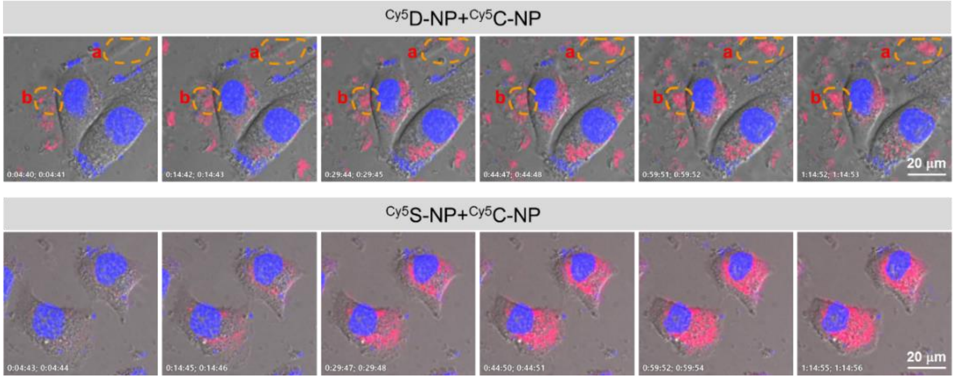

Supplementary Fig. 18 Series CLSM images of live 4T1 cells co-incubated with Cy5-labeled D-NP/C-NP or S-NP/C-NP. The Cy5-labeled D-NP/C-NP or S-NP/C-NP was pre-incubated for 2 h under pH 6.5. The formed particles aggregations could stay outside the cells in the ^{Cy5}D-NP/^{Cy5}C-NP group (a, b in the dashed ring), resulting in lower intracellular fluorescence signals. In contrast, higher intracellular fluorescence signals were in the ^{Cy5}S-NP/^{Cy5}C-NP group, and no obvious aggregations was formed outside the cells.

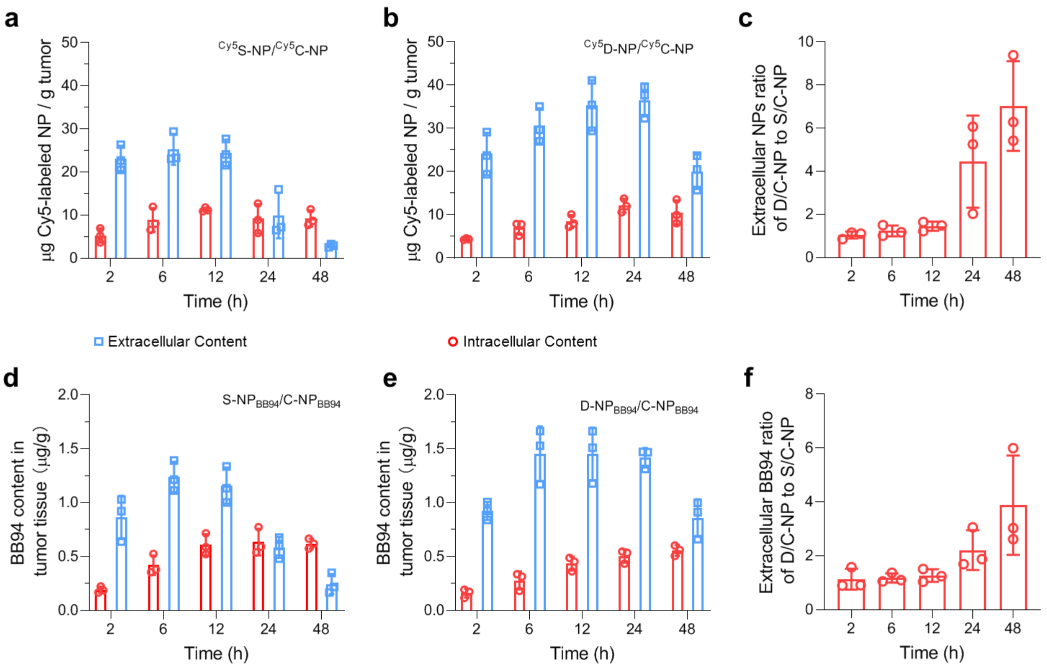

Supplementary Fig. 19 The extracellular or intracellular quantitative analysis of **a-c** nanoparticles and **d-f** BB94 in the tumor tissue after i.v. administration Cy5-labeled S-NP$_{BB94}$/C-NP$_{BB94}$ or D-NP$_{BB94}$/C-NP$_{BB94}$ by fluorescence spectrophotometer and HPLC. Data were presented as the mean ± s.d. (n = 3).

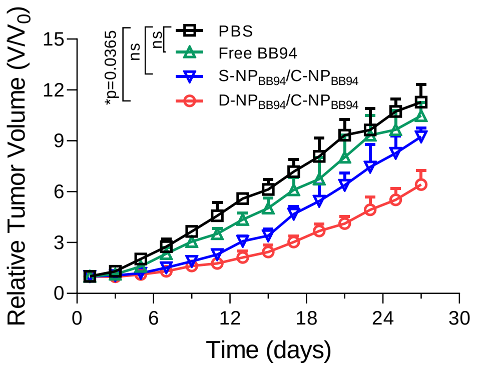

Supplementary Fig. 20 The average growth curves of orthotopic 4T1 breast tumor after different treatment in Fig. 4. Data were presented as the mean ± s.d. (n = 5). Statistical significance was calculated via one-way ANOVA with a Tukey post hoc test. *$p < 0.05$.

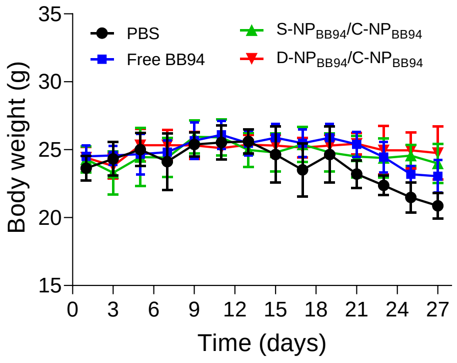

Supplementary Fig. 21 Balb/c mice bearing orthotopic 4T1 tumors were treated as indicated in Fig. 4a, the body weight changes of mice in the course of treatment. Data were presented as the mean ± s.d. n = 5 biological independent mice.

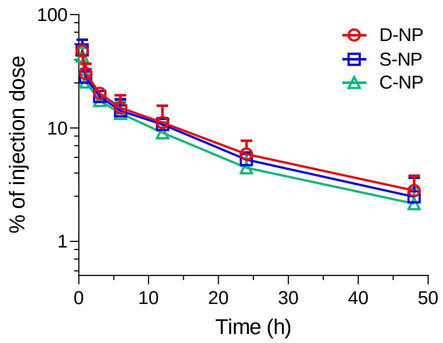

Supplementary Fig. 22 *In vivo* pharmacokinetic profiles after intravenous injection of Cy5-labeled D-NP, S-NP and C-NP. Data were presented as the mean ± s.d. (n = 3).

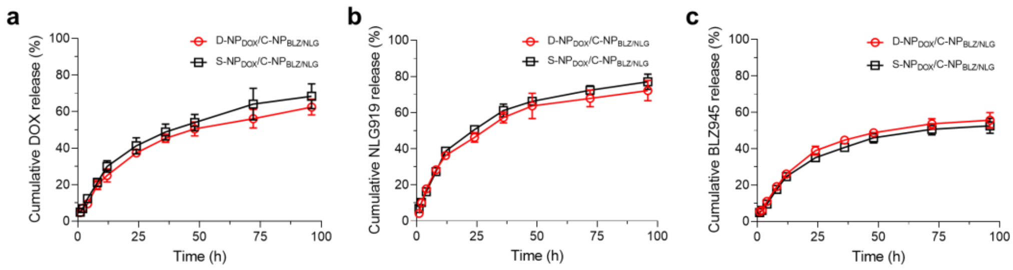

Supplementary Fig. 23 The release behavior of **a** DOX, **b** NLG919 and **c** BLZ945 from S-NP_{DOX}/C-$NP_{BLZ/NLG}$ and D-NP_{DOX}/C-$NP_{BLZ/NLG}$ under acidy environment (pH 6.5). Data were presented as the mean ± s.d. (n = 3).

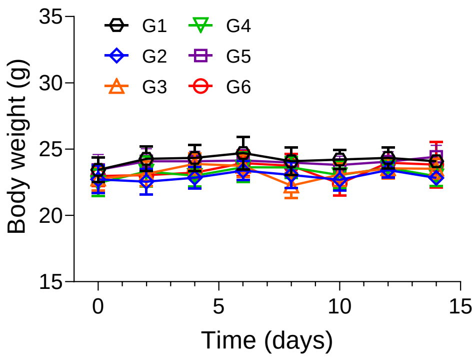

Supplementary Fig. 24 Balb/c mice bearing orthotopic 4T1 tumors were treated as indicated in Fig. 6b, the body weight changes of mice in the course of treatment. Data were presented as the mean ± s.d. n = 5 biological independent mice. G1: PBS, G2: DOX/BLZ945/NLG919, G3: D-NP_{DOX}, G4: D-NP_{DOX}/C-NP, G5: S-NP_{DOX}/C-$NP_{BLZ\&NLG}$, G6: D-NP_{DOX}/C-$NP_{BLZ\&NLG}$.

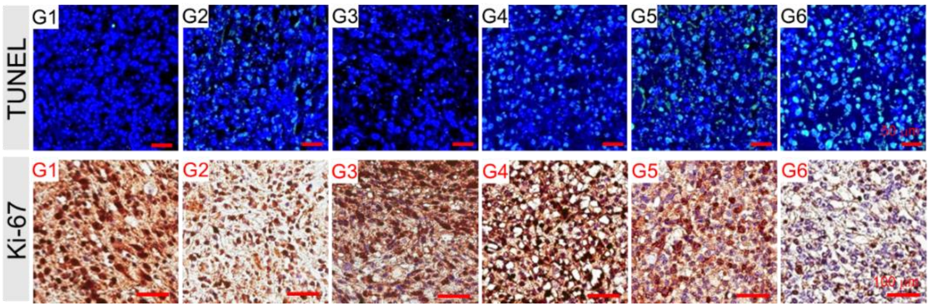

Supplementary Fig. 25 Tumor histological analysis with TUNEL (green, TUNEL; blue, DAPI) and Ki-67 (recombinant anti-Ki67 antibody, ab16667, 1:200 dilution) staining after different treatments. The representative image was from one of five independent fields of view in a single experiment. G1: PBS, G2: DOX/BLZ945/NLG919, G3: D-NP_{DOX}, G4: D-NP_{DOX}/C-NP, G5: S-NP_{DOX}/C-$NP_{BLZ\&NLG}$, G6: D-NP_{DOX}/C-$NP_{BLZ\&NLG}$. Scale bar, 100 μm.

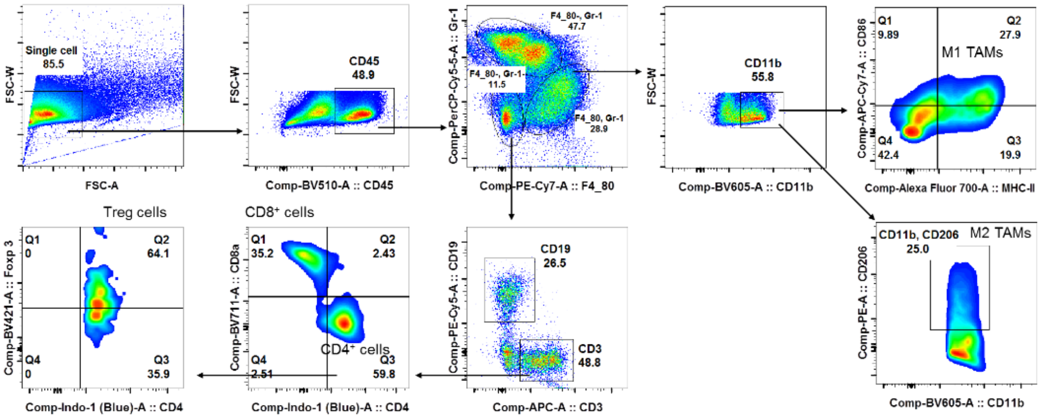

Supplementary Fig. 26 Gating strategy for immune cell analysis by flow cytometry. Immune cell population was gated based on the expression of CD45 (118138 events in gate), CD45$^+$ cells were further gated to determine F4/80$^-$Gr-1$^-$ lymphocyte cells (13734 events in gate), and F4/80$^+$Gr-1$^-$ macrophage cells (33703 events in gate). The F4/80$^-$Gr-1$^-$ lymphocyte cells were further gated to determine CD3$^+$ T cells (6697 events in gate), CD3$^+$CD8$^+$ (2358 events in gate), CD3$^+$CD4$^+$ T cells (4005 events in gate) and CD3$^+$CD4$^+$Foxp3$^+$ Treg cells (2568 events in gate). The F4/80$^+$Gr-1$^-$ cells were further gated to determine CD11b$^+$CD86$^+$MHC-II$^+$ M1 macrophages (3157 events in gate) and CD11b$^+$CD206$^+$ M2 macrophages (5768 events in gate). Cell populations were gated sequentially following arrows, and isotype controls were used for identifying the non-specific background.

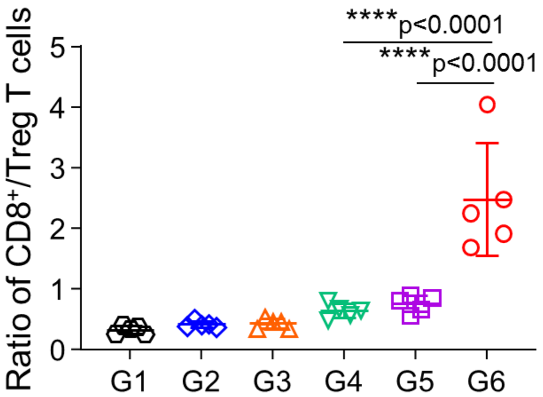

Supplementary Fig. 27 Ratio of CD3$^+$CD8$^+$ T cells to CD3$^+$CD4$^+$Foxp3$^+$ Treg cells in tumor after treated as indicated in Fig. 6b. G1: PBS, G2: DOX/BLZ945/NLG919, G3: D-NP$_{DOX}$, G4: D-NP$_{DOX}$/C-NP, G5: S-NP$_{DOX}$/C-NP$_{BLZ\&NLG}$, G6: D-NP$_{DOX}$/C-NP$_{BLZ\&NLG}$. Data are presented as mean ± s.d. (n = 5). Statistical significance was calculated via one-way ANOVA with a Tukey post-hoc test. ****p< 0.0001.

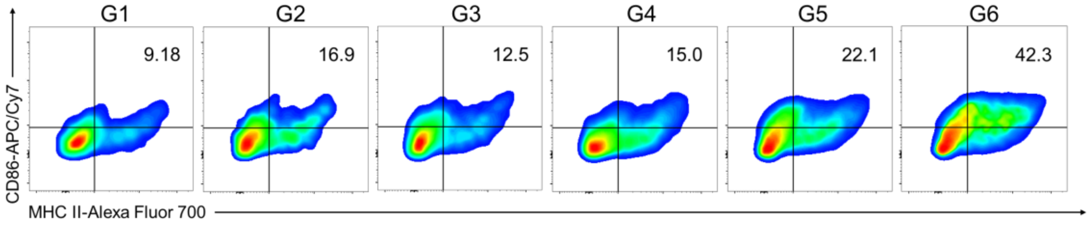

Supplementary Fig. 28 Representative flow cytometric analysis images of M1-like macrophages (CD86$^+$MHC-II$^+$) gating on CD45$^+$F4/80$^+$CD11b$^+$ cells. G1: PBS, G2: DOX/BLZ945/NLG919, G3: D-NP$_{DOX}$, G4: D-NP$_{DOX}$/C-NP, G5: S-NP$_{DOX}$/C-NP$_{BLZ\&NLG}$, G6: D-NP$_{DOX}$/C-NP$_{BLZ\&NLG}$.

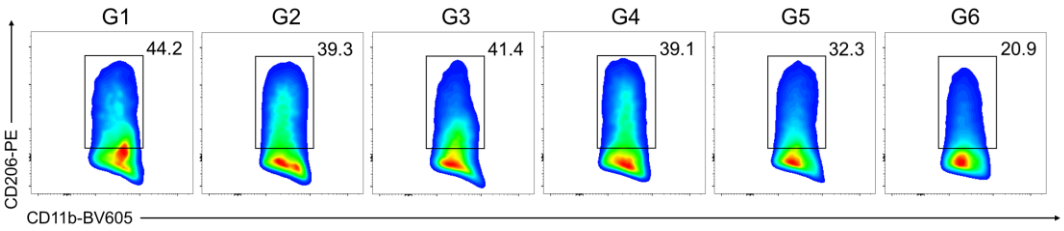

Supplementary Fig. 29 Representative flow cytometric analysis images of M2-like macrophages (CD206$^+$) gating on CD45$^+$F4/80$^+$CD11b$^+$ cells. G1: PBS, G2: DOX/BLZ945/NLG919, G3: D-NP$_{DOX}$, G4: D-NP$_{DOX}$/C-NP, G5: S-NP$_{DOX}$/C-NP$_{BLZ\&NLG}$, G6: D-NP$_{DOX}$/C-NP$_{BLZ\&NLG}$.

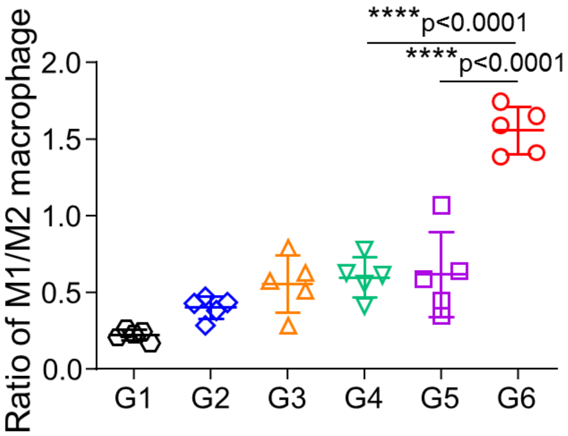

Supplementary Fig. 30 Ratio of CD11b$^+$CD86$^+$MHC-II$^+$ M1-like macrophages to CD11b$^+$CD206$^+$ M2-like macrophagse in tumor after treated as indicated in Fig. 6b. G1: PBS, G2: DOX/BLZ945/NLG919, G3: D-NP$_{DOX}$, G4: D-NP$_{DOX}$/C-NP, G5: S-NP$_{DOX}$/C-NP$_{BLZ\&NLG}$, G6: D-NP$_{DOX}$/C-NP$_{BLZ\&NLG}$. Data are presented as mean ± s.d. (n = 5). Statistical significance was calculated via one-way ANOVA with a Tukey post-hoc test. ****$p < 0.0001$.

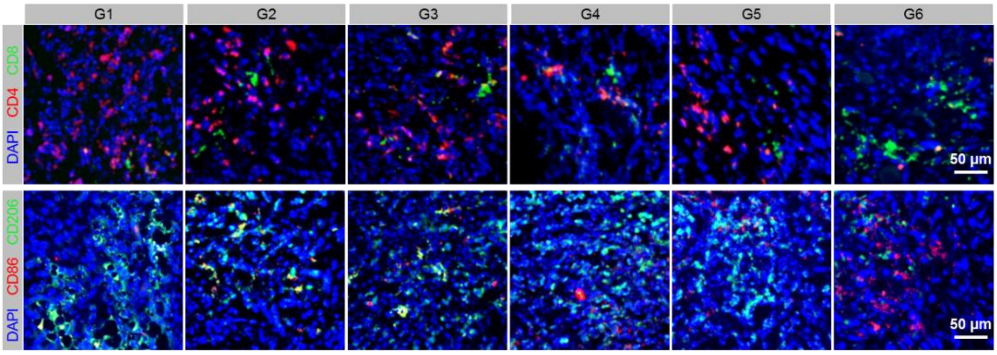

Supplementary Fig. 31 Representative immunofluorescence images of CD8 (green), CD4 (red) and CD206 (green) and CD86 (red) in tumor sections obtained at the end of different treatment in Fig. 6. The representative image was from one of five independent fields of view in a single experiment. G1: PBS, G2: DOX/BLZ945/NLG919, G3: D-NP$_{DOX}$, G4: D-NP$_{DOX}$/C-NP, G5: S-NP$_{DOX}$/C-NP$_{BLZ\&NLG}$, G6: D-NP$_{DOX}$/C-NP$_{BLZ\&NLG}$.

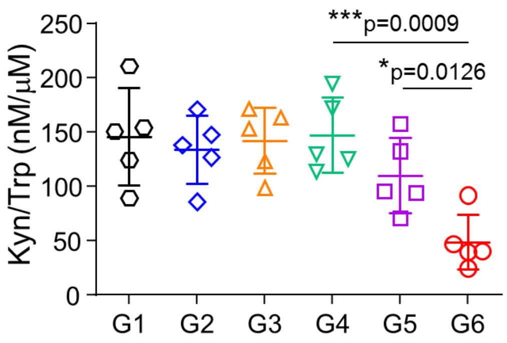

Supplementary Fig. 32 Intratumoral Kyn to Trp ratio examined after treated as indicated in Fig. 6b. G1: PBS, G2: DOX/BLZ945/NLG919, G3: D-NP$_{DOX}$, G4: D-NP$_{DOX}$/C-NP, G5: S-NP$_{DOX}$/C-NP$_{BLZ\&NLG}$, G6: D-NP$_{DOX}$/C-NP$_{BLZ\&NLG}$. Data are presented as mean ± s.d. (n = 5). Statistical significance was calculated via student's t-test (two tails). *$p < 0.05$, ***$p < 0.001$.

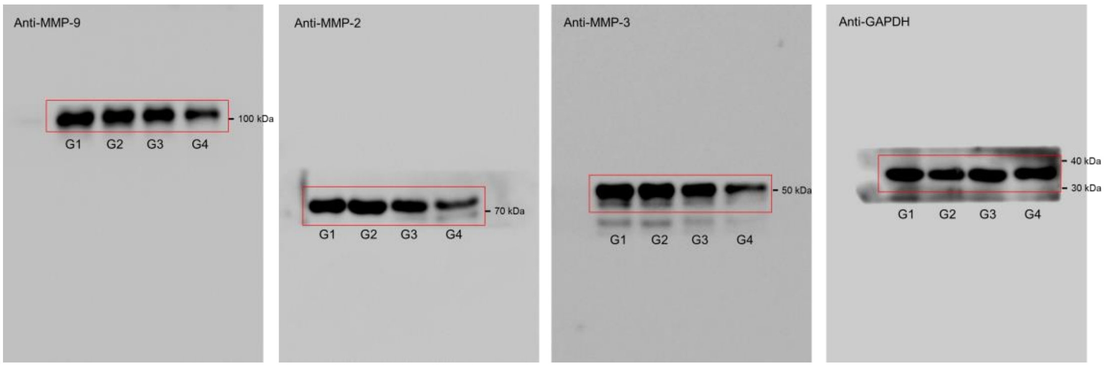

Supplementary Fig. 33 The uncropped versions of immunoblot images in Fig. 4d. All protein samples were prepared according to the manufacturer's instructions, separated by 12% SDS-PAGE and transferred to PVDF membranes using a wet-transfer system. The membranes were blocked in TBST buffer containing 5% (wt/vol) BSA for 2 h at room temperature and then were cut and incubated with different primary antibodies (Anti-GAPDH with 1:10000 dilution, Anti-MMP2 with 1:1000 dilution, Anti-MMP3 with 1:1000 dilution and Anti-MMP9 with 1:1000 dilution) diluted in TBST buffer containing 1% (wt/vol) BSA overnight at 4 °C, respectively. The membranes were further incubated with HRP-conjugated secondary antibody (goat anti-rabbit IgG H&L (HRP) with 1:2000 dilution and goat anti-mouse IgG H&L (HRP) with 1:2000 dilution) diluted in TBST buffer containing 1% (wt/vol) BSA for 1 h at room temperature. Finally, the membranes were developed using SuperSignal Chemiluminescent Substrates (Thermo Fisher Scientific, MA USA). G1: PBS, G2: Free BB94, G3: S-NP_{BB94}/C-NP_{BB94}, G4: D-NP_{BB94}/C-NP_{BB94}.

Supplementary Methods

Materials. Carboxylated polyethylene glycol (HOOC-PEG-OH, Mn=3400 g/mol) and tert-butoxycarbonyl protected amino polyethylene glycol (BocNH-PEG-OH, Mn=3400 g/mol) were purchased from Guangzhou Tansh-Tech Co., Ltd. DA-LA lactide was purchased from Jinan Daigang Biological Technology Co., Ltd. Cysteine and 1-(3-Dimethylaminopropyl)-3-ethylcarbodiimide hydrochloride (EDC·HCl) were purchased from Aladin Reagents Co., Ltd. Batimastat (BB-94) was purchased from MedChemExpress company. N,N'-Dicyclohexylcarbodiimide (DCC), 1-Hydroxybenzotriazole (HBOT), and N-hydroxysuccinimide (NHS) were purchased from Macklin Biochemical Technology Co., Ltd. Lipophilic fluorescent dyes DiD and DiI were purchased from Energy Chemical company. 2-Cyano-6-aminobenzothiazole (CBT) was purchased from Sigma-Aldrich Chemical Reagents Co., Ltd. Cy5-NHS was purchased from J&K Scientific Co., Ltd.

Tumor necrosis factor-α (TNF-α), interferon -γ (IFN-γ), interleukin-10 (IL-10) and interleukin-12p70 (IL-12p70) ELISA kits were purchased from Dakewe Biotech Co., Ltd. Recombinant anti-Ki67 antibody [SP6] (ab16667), recombinant Anti-GAPDH (ab181602), recombinant Anti-MMP3 (ab52915), goat anti-rabbit IgG H&L (HRP) (ab6721), goat anti-mouse IgG H&L (HRP) (ab6789), recombinant Anti-MMP2 (ab86607) and recombinant Anti-MMP9 (ab228402) were purchased from Abcam company.

Characterization. NMR spectra were recorded in deuterated reagent (such as, $CDCl_3$ or DMSO-d_6) with an NMR Bruker AVANCE III 400 MHz spectrometer (Bruker

Scientific Corporation Ltd., Switzerland). The size measurement was carried out in aqueous solution using a Malvern ZS90 dynamic light scattering instrument (Malvern Instruments Ltd., England) with a He-Ne laser (633 nm) and 90° collecting optics. The data were analyzed using Malvern Dispersion Technology Software 5.10. The fluorescence spectra were measured on a fluorescence spectrophotometer (Shimadzu RF-6000, Japan). Transmission electron microscopy (TEM) measurements were made using a JEM-2100F transmission electron microscopy (TEM) at an accelerating voltage of 200 kV.

Synthesis of tert-butoxycarbon protected cysteine (Boc-Cys). Cysteine (5 g, 0.029 mol), $NaHCO_3$ (2.436 g, 0.029 mol), 50 mL THF and 50 mL ultrapure water were added to a 100 mL clean round-bottomed flask and keep stirring until the solid dissolved. Then, Di-tert-butyl pyrocarbonate (Boc_2O, 6.322 g, 0.029 mol) was added dropwise at a low temperature of 0 °C and continued stirring for 30 min. After that, the reaction was naturally heated to room temperature and keep stirring overnight. The Boc-Cys crude product solution was concentrated by a rotary evaporator to remove THF, then collect clarified filtrate after filtration and adjust the filtrate's pH value to 8~9 with diluted hydrochloric acid. The product solution produced a mass of white precipitates and then continued stewing for 2 h. Further, the white product was filtered, dried overnight, and collected, yield the Boc-Cys.

Synthesis of Cys-PEG-*b*-PLA. First, tert-butoxycarbonyl protected amino polyethylene glycol-polylactic acid (tBocNH-PEG-*b*-PLA) was synthesized through a ring-opening polymerization (ROP) method in a glove box (H_2O < 0.01 ppm, O_2 <

0.01 ppm). Anhydrous BocNH-PEG-OH (Mn = 3400 g/mol, 1 g, 0.294 mmol) and anhydrous lactide (1.1 g, 7.639 mmol) were added into a 50 mL pre-baked dry round-bottom flask, and added 30 mL anhydrous toluene keep stirring to dissolve the solid reactant at 85 °C. Then, $Sn(Oct)_2$ (20 mg, 0.049 mmol) was added under stirring conditions and continue stirring for 3 h. After the reaction, the concentrated product was precipitated in a methanol/ether mixture (1/10, v/v), then filtered and dried to obtain BocNH-PEG-*b*-PLA. Subsequently, BocNH-PEG-*b*-PLA was dissolved in a trifluoroacetic acid/tetrahydrofuran mixture (20 mL, TFA/THF, 9/1, V/V) and stirred under nitrogen protection for 3 h at room temperature. Then, the concentrated product was also precipitated in the cold methanol/ether mixture (1/10, v/v), filtered, and dried under vacuum conditions to obtain H_2N-PEG-*b*-PLA.

Furthermore, the Boc-Cys-PEG-*b*-PLA was synthesized by an amidation of H_2N-PEG-*b*-PLA and Boc-Cys. Boc-Cys (0.126 g, 0.57 mmol), 1-hydroxybenzotriazole (HOBT, 0.145 g, 0.684 mmol) and 1-ethyl-(3-dimethylaminopropyl) carbodiimine hydrochloride (EDC·HCl, 0.131 g, 0.684 mmol) were added into a 10 mL round-bottomed flask contained 10 mL DCM and keep stirring for 6 h at room temperature. Subsequently, H_2N-PEG-*b*-PLA (1.1 g, 0.162 mmol) was pre-dissolved in 10 mL DCM and then quickly poured into the above-mixed reaction solution and continue stirring for 24 h at 25°C. After the reaction, the concentrated product solution was precipitated in the cold methanol/ether mixture (1/10, v/v). The solution was then filtered, vacuum drying overnight to obtained faint yellow Boc-Cys-PEG-*b*-PLA. Then, Boc-Cys-PEG-*b*-PLA was

dissolved in TFA/THF mixture (20 mL, 9/1, V/V) and stirred at room temperature for 3 h under nitrogen protection to slough the Boc protection. After the same precipitation process as mentioned above, Cys-PEG-*b*-PLA was obtained.

Synthesis of CBT-PEG-*b*-PLA. First, HOOC-PEG-*b*-PLA was synthesized by ring-opening polymerization in a glove box (H_2O < 0.01 ppm, O_2 < 0.01 ppm). Anhydrous HOOC-PEG-*b*-PLA (Mn = 3400g/mol, 1 g, 0.294 mmol), anhydrous lactide (1.1 g, 7.639 mmol), and 30 mL anhydrous toluene were added to a dry 50 mL round-bottomed flask, keep stirring to dissolve the solid reactants at 85 °C. Then, $Sn(Oct)_2$ (20 mg, 0.049 mmol) was added under stirring conditions and continue stirring for 3 h.

At the end of the reaction, the concentrated product solution was precipitated in the cold methanol/ether mixture (1/10, v/v), filtered, and vacuum dried overnight to obtain yellow HOOC-PEG-*b*-PLA. Then, CBT-PEG-*b*-PLA was synthesized through amidation reaction: HOOC-PEG-*b*-PLA (1.1 g, 0.162 mmol), EDC·HCl (37.2 mg, 0.194 mmol) and HOBT (41.2 mg, 0.194 mmol) were added into a 50 mL round-bottom flask with magnetons, and then 10 mL DCM was added to dissolve the above solid reactants and keep stirring for 6 h at room temperature. Then, CBT (34.2 mg, 0.195 mmol) was added into the above-mixed reaction solution and continued stirring for 24 h at room temperature. After the reaction, the concentrated product solution was then dropped into a cold methanol/ether mixture (1/10, v/v). Then, the yellow CBT-PEG-*b*-PLA solid product was obtained after filtration and vacuum drying overnight.

DA or SA masked cysteine residues of Cys-PEG-*b*-PLA. 2, 3-dimethylmaleic anhydride (DA) and succinic anhydride (SA) were used to block the amino group of cysteine residues at the terminal of Cys-PEG-*b*-PLA and obtained DA-Cys-PEG-*b*-PLA and SA-Cys-PEG-*b*-PLA, respectively. 200 mg Cys-PEG-*b*-PLA polymer material was dissolved in THF, and blank Cys-NP nanoparticles were prepared by the nanoprecipitation method. Then, five times cysteine residue amino equivalent DA or SA were added to the blank Cys-NP solution (2 mg/mL, 50 mL) in batches. The pH value of the solution was adjusted to 8-9 by NaOH solution (1.0 M) and continued to stir at room temperature for 4 h. After the reaction, the nanoparticle solution was concentrated and purified by ultrafiltration with a YM-30 ultrafiltration centrifugal tube (Millipore, MWCO 3000Da), and the resulting nanoparticles were denoted as D-NP and S-NP, respectively. Finally, the two nanoparticle solutions were freeze-dried to obtain DA-Cys-PEG-*b*-PLA and SA-Cys-PEG-*b*-PLA.

Preparation of Cy5 labeled ^{Cy5}D-NP, ^{Cy5}S-NP and ^{Cy5}C-NP. First, Cy5-PEG-*b*-PLA was synthesized: Cy5-NHS (61 mg, 0.081 mmol) and H_2N-PEG-*b*-PLA (0.5 g, 0.074 mmol) were added into a round-bottom flask (25 mL) with a stir magneton. Then, 10 mL THF was added into the flask and continue stirring at room temperature for 8 h under dark. At the end of the reaction, concentrate the above reaction product solution to ~2 mL and drop it into the cold methanol/ether mixed solution (1/10, v/v). The product precipitation was filtered and dried to obtain Cy5-PEG-*b*-PLA. The preparation of Cy5-labeled D-NP nanoparticles was as follows: 10 mg

DA-Cys-PEG-*b*-PLA and 1 mg Cy5-PEG-***b***-PLA were dissolved in 1 mL DMSO. After vortex, the mixed materials solution was dripped into PBS (10 mL, 0.01 M, pH 7.4) and keep stirring for 2 h at R.T. The obtained nanoparticles solution was transferred into a dialysis bag (MWCO = 14000 Da) and put into PBS (0.01 M, pH 7.4, 2 L) for dialysis overnight. Then, collect the solution inside the dialysis bag and concentrate it by a YM-30 ultrafiltration centrifuge tube (Millipore, MWCO 5000 Da), and the obtained nanoparticles were denoted as ^{Cy5}D-NP. Similarly, ^{Cy5}C-NP or ^{Cy5}S-NP nanoparticles were prepared in the same process as above with using CBT-PEG-*b*-PLA or SA-Cys-PEG-*b*-PLA replace DA-Cys-PEG-*b*-PLA in the preparation process.

Preparation of FITC labeled ^{FITC}C-NP. The preparation of FITC-labeled ^{FITC}C-NPs was as follows. First, FITC (68.53 mg, 0.176 mmol), 1-ethyl-3-(3-dimethylaminopropyl)carbodiimide hydrochloride (EDC·HCl) (37.2 mg, 0.194 mmol) and N-hydroxysuccinimide (NHS) (22.3 mg, 0.194 mmol) were dissolved in 10 mL of tetrahydrofuran (THF) and stirred at R.T. for 6 h. Then, H_2N-PEG-*b*-PLA (1.0 g, 0.147 mmol) was added to the reaction solution, and stirring was continued at R.T. for 24 h. After the reaction, the product solution was concentrated and added dropwise to a mixed solution of diethyl ether/methanol (10/1, v/v, 100 mL), filtered and dried to obtain FITC-PEG-*b*-PLA. Subsequently, 1.1 mL of DMSO containing 10 mg of CBT-PEG-*b*-PLA and 1 mg of FITC-PEG-*b*-PLA were added dropwise into PBS (10 mL, 0.01 M, pH 7.4) and stirred for 2 h to obtain ^{FITC}C-NPs. Then, the ^{FITC}C-NPs were transferred to a dialysis bag (MWCO 14000 Da)

for dialysis overnight and concentrated by using a YM-30 ultrafiltration centrifuge tube (Millipore, MWCO 5000 Da).

Preparation of BB94-loaded nanoparticles. BB94 loaded nanoparticles D-NP_{BB94}, S-NP_{BB94} and C-NP_{BB94} were prepared by the single emulsification method. Typically, DA-Cys-PEG-*b*-PLA, SA-Cys-PEG-*b*-PLA or CBT-PEG-*b*-PLA (10 mg in 500 μL dichloromethane), BB94 (1.5 mg in 100 μL dichloromethane) and 6 mL ultrapure water were emulsified in a 50 mL centrifuge tube for 2 min at 65 W over an ice bath using a Vibra-cell VCX130 (Sonics & Materials, Inc., Newtown, CT, USA). Then, dichloromethane was removed via rotary evaporation at room temperature using a Rotavapor R-3 from Buchi Co. (New Castle, DE, USA). Subsequently, the obtained BB94-loaded nanoparticles were ultrafiltered, concentrated with YM-30 ultrafiltration centrifuge tube (Millipore, MWCO 3000 Da) and denoted as D-NP_{BB94}, S-NP_{BB94} and C-NP_{BB94}, respectively. The encapsulation efficiency of BB94 was measured by high-performance liquid chromatography (HPLC) analysis, using a Waters HPLC system equipping with a Waters 1525 binary pump, a Waters 2487 UV/visible detector, 1500 column heater, and a Symmetry C18 column. HPLC grade acetonitrile/water (80/20, v/v) was used as the mobile phase at 30 °C with a flow rate of 0.5 mL/min. The UV/visible detector was set at 285 nm and linked to Breeze software for data analysis. Linear calibration curves for concentrations in the range of 0.1-10.0 μg/mL were constructed using the absorbance by linear regression analysis. The concentration of BB94 in the solution was calculated based the standard curve. The loading contents of BB94 for C-NP_{BB94}, D-NP_{BB94} and S-NP_{BB94} were 3.78 ± 0.55%,

3.69 ± 0.41% and 3.55 ± 0.24%, respectively.

Preparation of DOX or BLZ945/NLG919-loaded nanoparticles. First, D-NP$_{DOX}$ and S-NP$_{DOX}$ were prepared by a nanoprecipitation method. 10 mg DA-Cys-PEG-*b*-PLA or SA-Cys-PEG-*b*-PLA and 1 mg DOX were dissolved in DMSO, then dropped into 10 mL PBS (pH 7.4, 0.01 M) and obtained D-NP$_{DOX}$ or S-NP$_{DOX}$, respectively. Subsequently, the D-NP$_{DOX}$ or S-NP$_{DOX}$ were transferred into a dialysis bag (MWCO 14000 Da) and dialyzed in PBS (pH 7.4, 0.01 M, 2 L) overnight. The nanoparticle solution was then concentrated by ultrafiltration with a YM-30 ultrafiltration centrifugal tube (Millipore, MWCO 3000Da). The encapsulation efficiency of DOX of D-NP$_{DOX}$ and S-NP$_{DOX}$ was measured by a UV spectrophotometer (UV-2600, Shimadzu, Japan). The loading contents of DOX for S-NP$_{DOX}$ and D-NP$_{DOX}$ were 4.76 ± 0.51% and 4.89 ± 0.45%, respectively.

Furthermore, The NLG919 and BLZ945 co-loaded C-NP were prepared by the single emulsification method. Typically, CBT-PEG-*b*-PLA (10 mg in 500 μL dichloromethane) puls BLZ945+NLG919 (1 mg + 1 mg in 200 μL dichloromethane) were transferred into the bottom of a 50 mL centrifuge tube, and then 6 mL ultrapure water was added and ultrasound for 2 min at 65 W over an ice bath using a Vibra-cell VCX130 (Sonics & Materials, Inc., Newtown, CT, USA). Then, dichloromethane was removed via rotary evaporation at room temperature using a Rotavapor R-3 from Buchi Co. (New Castle, DE, USA). Subsequently, the obtained drug-loaded nanoparticles were ultrafiltered, concentrated with YM-30 ultrafiltration centrifuge tube (Millipore, MWCO 3000 Da) and denoted as C-NP$_{NLG\&BLZ}$. The encapsulation

efficiency of BLZ945 or NLG919 was measured by high-performance liquid chromatography (HPLC) analysis, using a Waters HPLC system equipping with a Waters 1525 binary pump, a Waters 2487 UV/visible detector, 1500 column heater, and a Symmetry C18 column. HPLC grade acetonitrile/water (50/50, v/v) was used as the mobile phase at 30 °C with a flow rate of 0.5 mL/min. The UV/visible detector was set at 280 nm and linked to Breeze software for data analysis. Linear calibration curves for concentrations in the range of 0.1-10.0 μg/mL were constructed using the absorbance by linear regression analysis. The concentration of BLZ945 or NLG919 in the solution was calculated based on the standard curve. The loading content of NLG 919 and BLZ945 was 5.31 ± 0.36% and 4.93 ± 0.52%, respectively.

Drug release test *in vitro*. The BB94 release profiles from nanoparticles in different pH was tested by dialysis method. The dialysis bags (MWCO: 14 kDa) containing 1 mL of D-NP_{BB94}/C-NP_{BB94} or S-NP_{BB94}/C-NP_{BB94} (BB94 concentrations was 90 μg/mL) were immersed into 50 mL tubes containing 15 mL PB buffer (0.02 M, pH 6.5 or 7.4) and these tubes were put into incubator sharker (37 °C). The external PB buffer was collected and replaced with equal volume of fresh PB buffer at different periods. The collected solutions were freeze-dried and redissolved in acetonitrile, centrifuged to determine the concentration of BB94 by HPLC with similar method as abovementioned.

The DOX, BLZ945 and NLG919 release profiles from D-NP_{DOX}/C-$NP_{BLZ\&NLG}$ or S-NP_{DOX}/C-$NP_{BLZ\&NLG}$ were tested by similar abovementioned dialysis method, with DOX, BLZ945 and NLG919 concentrations inside dialysis bags were 92.6, 80.0 and

72.6 μg/mL, respectively. The collected solutions were freeze-dried and redissolved in acetonitrile, centrifuged to determine the concentration of DOX by fluorospectro photometer, while BLZ945 and NLG919 determined by HPLC with abovementioned method.

Cellular uptake experiments *in vitro*. 4T1 cells were seeded in 24-well plates with 2×10^4 cells per well in 0.5 mL RPMI 1640 (containing 10% FBS) and incubated in a humidified 5% CO_2 atmosphere for 12 h. ^{Cy5}D-NP/^{Cy5}C-NP mixed solution ([^{Cy5}D-NP] = 1 mg/mL, [^{Cy5}C-NP] = 1 mg/mL) was pre-stirred at pH 6.5 for different times (0.5 h, 1 h, 2 h and 4 h), and then co-incubated with 4T1 cells at a final concentration of 0.1 mg/mL for 2 h. The ^{Cy5}D-NP/^{Cy5}C-NP pre-stirred at pH 7.4 for 2 h was used as a control group. After incubation, the cells washed with 1×PBS removed extracellular particles and trypsinized for FACS to detect the intracellular Cy5 fluorescence intensity.

For the real-time observation of uptake behavior, 4T1 cells seeded in a glass-bottom cell culture dish (ϕ15 mm) at a density of 5×10^4 cells per dish and incubated with 1 mL RPMI 1640 (containing 10% FBS) in a humidified 5% CO_2 atmosphere for 12 h. The nucleus of live 4T1 cells was pre-stained with Hochest 33342 (1:100 dilution, Thermo Scientific™) for 5min. Then, the culture dish was fixed on the objective table of CLSM and selected an appropriate field of live cells vision. Subsequently, the mixed nanoparticles (0.1 mg/mL, ^{Cy5}D-NP/^{Cy5}C-NP or ^{Cy5}S-NP/^{Cy5}C-NP were pre-stirred at pH 6.5 for 2 h) were added into the culture dish. Then, the live 4T1 cell uptake behavior was observed by continuous confocal imaging for 75 min (Olympus,

IXplore SpinSR, Cellsens software v2.2).

Plasma clearance and biodistribution. First, the platinum prodrug was synthesized according to previous reports[1]. Then, the platinum-loaded D-NP, S-NP and C-NP were obtained through a nanoprecipitation method and the platinum drug-loading capacity was 2.17%, 2.45% and 2.31% determined by ICP-MS, respectively. Then, BALB/c female mice bearing 4T1 orthotopic tumors (n = 3 per group, 7 weeks) were intravenously administrated with platinum prodrug-loaded D-NP/C-NP or S-NP/C-NP at a dose of 40 μg/mouse of platinum. The blood was collected from the inferior vena cava using heparinized syringes and centrifuged to obtain the plasma at different defined time points (6 h, 12 h, 24 h, 48 h and 96 h). At the same time, the heart, liver, spleen, lung, kidney and tumor were excised and washed with PBS. All samples were weighed, and then acid digestion was performed with 80% HNO_3 (4 mL concentrated HNO_3 and 1 mL 30% w/w H_2O_2). The platinum concentration was measured by ICP-MS (iCAP RQ, Thermofisher Scientific) after constant the samples volume to 50 mL with the ultrapure water.

Moreover, we used Cy5 covalently labeled D-NP/C-NP and S-NP/C-NP and BALB/c female mice bearing 4T1 orthotopic tumors (n = 6 per group, 7 weeks) were intravenously administrated with ^{Cy5}D-NP/^{Cy5}C-NP or ^{Cy5}S-NP/^{Cy5}C-NP at a dose of 2 mg of NPs per mouse. At 24 h and 96 h post-injection, three mice were randomly selected from each group and sacrificed. The major organ (heart, liver, spleen, lung and kidney) and tumor tissues were excised, washed with cold saline, dried with filter paper, weighed, and put into 2 mL FastPrep® homogenization tubes with three

stainless steel beads (2 mm), and 1mL of methanol was added. Subsequently, the tumor tissue samples were homogenized at 9391 g for 2 min, performed with 10 s of homogenization at 10 s intervals. After centrifugation (10000 g, 10 min), the NPs in the supernatant were examined with a fluorescence spectrophotometer (Cytation™ 5, BioTek, Winooski, VT, USA). Standard curves of ^{Cy5}D-NP/^{Cy5}C-NP, ^{Cy5}S-NP/^{Cy5}C-NP solutions (0.8, 1.6, 3.125, 6.25, 12.5, 25, 50 and 100 μg/mL) were constructed.

The quantitative experiment *in vivo*. BALB/c female mice bearing 4T1 orthotopic tumors (n = 3 per group, 7 weeks) were intravenously administrated with BB94-loaded ^{Cy5}D-NP/^{Cy5}C-NP or ^{Cy5}S-NP/^{Cy5}C-NP at a dose of 100 μg/mouse of BB94 (the dose of particles were 2.94 mg/mouse and 2.81 mg/mouse, respectively). At different defined time points (2 h, 6 h, 12 h, 24 h and 48 h), the tumor tissues were excised and washed with PBS. Then, the tumor tissues were processed through mechanical disruption and enzymatically digested and filtered to obtain single-cell suspensions according to the well-established protocol[2]. The cell suspensions were centrifuged to harvest the extracellular space solution and cells with 376 g, 5 min. The obtained cell precipitates further were lysed by using 1% Triton X-100 in PBS (1 mL) with a homogenizer, followed by three freeze-thaw cycles and further centrifuged to obtain intracellular space solution. Finally, the particles in the extracellular or intracellular space were examined with a fluorescence spectrophotometer (Cytation™ 5, BioTek, Winooski, VT, USA). Standard curves of ^{Cy5}D-NP/^{Cy5}C-NP and ^{Cy5}S-NP/^{Cy5}C-NP solutions (0.8, 1.6, 3.125, 6.25, 12.5, 25, 50 and 100 μg/mL) were constructed. For the extracellular/intracellular BB94 content detection, the

abovementioned obtained solutions were extracted with chloroform/acetonitrile component solvent (1 mL, 4/1, v/v) on a vortex mixed for 5 min. Following centrifugation at a speed of 10000 g for 20 min, the organic phase was obtained and dried under vacuum condition, further dissolved in 1 mL acetonitrile (HPLC). The concentration of extracellular/intracellular BB94 was measured by high-performance liquid chromatography (HPLC) analysis with the abovementioned method.

Pharmacokinetic study. Female ICR mice were randomly divided into three groups (n=3 per group, 8 weeks). Mice were intravenously injected with Cy5-labeled D-NP, C-NP or S-NP, respectively. At various time points post-administration, blood samples were collected from the retro-orbital plexus of the eye and then placed in heparinized (1000 U/mL) tubes and centrifuged (9391 g, 10 min) to obtain plasma. After centrifugation, the concentration of Cy5-labeled NPs in the supernatant was analyzed by a fluorescence spectrophotometer (Cytation™ 5, BioTek, Winooski, VT, USA; Ex: 620 nm, Em: 650 nm).

Immunohistochemical staining. Tumor sections (8 μm) were treated with sodium citrate buffer (10 mM sodium citrate and 0.05% Tween 20, pH 6.0) for 10 min at 90 °C for antigen retrieval after hydration. Prior to staining, the tissue sections on slides were blocked with 5% (v/v) normal goat serum (Gibco) and incubated with primary antibodies against iNOS (ab49999, 1/1000 dilution) or Arg-1 (ab96183, 1/500 dilution) overnight at 4 °C. Next, the sections were washed three times with PBS and incubated for 1 h with Alexa Fluor® 647-conjugated (ab150115, 1/1000 dilution) or Alexa Fluor® 488-conjugated (ab150077, 1/1000 dilution) secondary antibodies.

After washing with PBS, the slides were mounted with mounting medium (VectaMount mounting medium, Vector Laboratories Inc., Burlingame, CA) and imaged by CLSM (Zeiss, LSM880).

For the infiltrating immune cells staining, 20 μg/mL FITC anti-mouse CD8a antibody (Biolegend, cat no. 100706, clone: 53-6.7, 1/500 dilution) and PE/Dazzle^{TM} 594 anti-mouse CD4 antibody (Biolegend, cat no. 100456, clone: GK1.5, 1/500 dilution) was used to stain the tumor slices overnight at 4 °C, while 20 μg/mL APC anti-mouse CD86 antibody (Biolegend, cat no. 105012, clone: GL-1, 1/500 dilution) and PE anti-mouse CD206 antibody (Biolegend, cat no. 141706, clone: C068C2, 1/500 dilution) was used to stain the tumor slices overnight at 4 °C for macrophages staining.

Supplementary References

1. Dhar, S., Gu, F. X., Langer, R., Farokhzad, O. C. & Lippard, S. J. Targeted delivery of cisplatin to prostate cancer cells by aptamer functionalized Pt(IV) prodrug-PLGA-PEG nanoparticles. *Proc. Natl. Acad. Sci. USA* **105**, 17356-17361 (2008).
2. Yang, Z. J. et al. Tumor-killing nanoreactors fueled by tumor debris can enhance radiofrequency ablation therapy and boost antitumor immune responses. *Nat. Commun.* **12**, 4299 (2021).
