# Supplementary material for: Bioorthogonal in situ assembly of nanomedicines as drug depots for extracellular drug delivery
Source: Nat Commun. 2022 Apr 19;13:2038. doi: 10.1038/s41467-022-29693-8 (PMC9018704; doi:10.1038/s41467-022-29693-8)
Supplement: Supplementary file 3 — Description of Additional Supplementary Information [file 41467_2022_29693_MOESM3_ESM.pdf]

Description of Additional Supplementary Information

Supplementary Fig. 1 The synthetic route of **a** Boc-Cys and **b** Cys-PEG-*b*-PLA polymer.

Supplementary Fig. 2 The synthetic route of CBT-PEG-*b*-PLA polymer.

Supplementary Fig. 3 ^{1}H NMR spectra of Boc-Cys in DMSO-d_6.

Supplementary Fig. 4 ^{1}H NMR spectra of Boc-Cys-PEG-*b*-PLA in DMSO-d_6.

Supplementary Fig. 5 ^{1}H NMR spectra of Cys-PEG-*b*-PLA in DMSO-d_6.

Supplementary Fig. 6 ^{1}H NMR spectra of CBT-PEG-*b*-PLA in DMSO-d_6.

Supplementary Fig. 7 The synthetic route of DA-Cys-PEG-*b*-PLA.

Supplementary Fig. 8 ^{1}H NMR spectra of DA-Cys-PEG-*b*-PLA in DMSO-d_6.

Supplementary Fig. 9 The stability of D-NP, C-NP and the mixed D-NP/C-NP in PBS solution containing 10% FBS.

Supplementary Fig. 10 ^{1}H NMR spectra of crosslinked blank D-NP and C-NP.

Supplementary Fig. 11 The synthetic route and ^{1}H NMR spectra of of SA-Cys-PEG-*b*-PLA.

Supplementary Fig. 12 The stability of S-NP and the mixed S-NP/C-NP in PBS solution containing 10% FBS.

Supplementary Fig. 13 ^{1}H NMR spectra of synthetic hydrophobic platinum prodrug (IV) in DMSO-d_6.

Supplementary Fig. 14 Pharmacokinetics curve in blood and biodistribution of platinum prodrug-loaded S-NP/C-NP or D-NP/C-NP after i.v. administration to BALB/c mice bearing an orthotopic 4T1 tumor.

Supplementary Fig. 15 The biodistribution of Cy5-labeled D-NP/C-NP and S-NP/C-NP in major organ and tumor at 48 h or 96 h i.v. post-injection.

Supplementary Fig. 16 The release behavior of encapsulated BB94 from S-NP_{BB94}/C-NP_{BB94} and D-NP_{BB94}/C-NP_{BB94}.

Supplementary Fig. 17 *In vitro* assembly of D-NP/C-NP decrease the cellular uptake.

Supplementary Fig. 18 Series CLSM images of live 4T1 cells co-incubated with

Cy5-labeled D-NP/C-NP or S-NP/C-NP.

Supplementary Fig. 19 The extracellular/intracellular NPs or BB94 quantitative analysis experiment.

Supplementary Fig. 20 The average growth curves of orthotopic 4T1 breast tumor after different treatment in Fig. 4.

Supplementary Fig. 21 Balb/c mice bearing orthotopic 4T1 tumors were treated as indicated in Fig. 4a.

Supplementary Fig. 22 *In vivo* pharmacokinetic profiles of D-NP, S-NP and C-NP.

Supplementary Fig. 23 The release behavior of DOX, NLG919 and BLZ945 from drug-loaded nanoparticles.

Supplementary Fig. 24 The body weight changes of mice in the treatment of Fig. 6b.

Supplementary Fig. 25 Tumor histological analysis with TUNEL and Ki-67 staining after different treatments.

Supplementary Fig. 26 Gating strategy for immune cell analysis by flow cytometry.

Supplementary Fig. 27 Ratio of $CD8^+$ T cells to Treg cells in tumor after treated as indicated in Fig. 6b.

Supplementary Fig. 28 Representative flow cytometric analysis images of M1-like macrophages.

Supplementary Fig. 29 Representative flow cytometric analysis images of M2-like macrophages.

Supplementary Fig. 30 Ratio of M1-like macrophages to M2-like macrophagse in tumor after treated as indicated in Fig. 6b.

Supplementary Fig. 31 Representative immunofluorescence images of CD8, CD4, CD206 and CD86 in tumor sections.

Supplementary Fig. 32 Intratumoral Kyn to Trp ratio examined after treated as indicated in Fig. 6b.

Supplementary Fig. 33 The uncropped versions of immunoblot images in Fig. 4d.

Supplementary Movie 1: Intravital CLSM real-time visualization movie of Cy5-labeled D-NP/C-NP accumulation in GFP-4T1 tumor-bearing mice.

Supplementary Movie 2: Intravital CLSM real-time visualization movie of Cy5-labeled S-NP/C-NP accumulation in GFP-4T1 tumor-bearing mice.

Supplementary Movie 3: Real-time CLSM movie of live 4T1 cells co-incubated with Cy5-labeled D-NP/C-NP or S-NP/C-NP.
