# Supplementary material for: Bioorthogonal in situ assembly of nanomedicines as drug depots for extracellular drug delivery
Source: Nat Commun. 2022 Apr 19;13:2038. doi: 10.1038/s41467-022-29693-8 (PMC9018704; doi:10.1038/s41467-022-29693-8)
Supplement: Supplementary file 7 — Reporting summary [file 41467_2022_29693_MOESM7_ESM.pdf]

nature portfolio

Corresponding author(s): Xianzhu Yang

Last updated by author(s): Mar 8, 2022

Reporting Summary

Nature Portfolio wishes to improve the reproducibility of the work that we publish. This form provides structure for consistency and transparency in reporting. For further information on Nature Portfolio policies, see our Editorial Policies and the Editorial Policy Checklist.

Statistics

For all statistical analyses, confirm that the following items are present in the figure legend, table legend, main text, or Methods section.

n/a	Confirmed	
☐	☒	The exact sample size (*n*) for each experimental group/condition, given as a discrete number and unit of measurement
☐	☒	A statement on whether measurements were taken from distinct samples or whether the same sample was measured repeatedly
☐	☒	The statistical test(s) used AND whether they are one- or two-sided<br>*Only common tests should be described solely by name; describe more complex techniques in the Methods section.*
☒	☐	A description of all covariates tested
☐	☒	A description of any assumptions or corrections, such as tests of normality and adjustment for multiple comparisons
☐	☒	A full description of the statistical parameters including central tendency (e.g. means) or other basic estimates (e.g. regression coefficient) AND variation (e.g. standard deviation) or associated estimates of uncertainty (e.g. confidence intervals)
☐	☒	For null hypothesis testing, the test statistic (e.g. *F*, *t*, *r*) with confidence intervals, effect sizes, degrees of freedom and *P* value noted<br>*Give P values as exact values whenever suitable.*
☒	☐	For Bayesian analysis, information on the choice of priors and Markov chain Monte Carlo settings
☒	☐	For hierarchical and complex designs, identification of the appropriate level for tests and full reporting of outcomes
☒	☐	Estimates of effect sizes (e.g. Cohen's *d*, Pearson's *r*), indicating how they were calculated

Our web collection on statistics for biologists contains articles on many of the points above.

Software and code

Policy information about availability of computer code

Data collection	NMR Bruker AVANCE III 400 MHz spectrometer software (Bruker Scientific Corporation Ltd., Switzerland); Malvern ZS90 dynamic light scattering instrument and Zetasizer software (version 7.12, Malvern Instruments Ltd., England); Fluorescence spectrophotometer software (Shimadzu RF-6000, Japan); Transmission electron microscopy (TEM) software (JEM-2100F, Japan); Flow cytometry: BD LSRFortessa and FACSDiva Software (version 6.0), BD AccuriTM C6 Plus and C6 Plus software (version 1.0.23.1); Data recording: Microsoft Excel 2016; Confocal microscopy: ZEISS ZEN microscope software (version 2.1), Nikon A1RMP confacal microscope software and Olympus IXplore SpinSR confacal microscope software (version 2.3); Living fluorescent imaging: Xenogen IVIS® Lumina system software (Caliper Life Sciences, USA); High-performance liquid chromatography (HPLC) analysis software (Breeze, version 7.4, Waters Alliance); Gel permeation chromatography (GPC) analysis software (version 6.2, Waters 1515 GPC). Highly sensitive series triple quadrupole mass spectrometer analysis software (SCIEX QTRAP 4500, USA). Inductively coupled plasma mass spectrometry analysis software (iCAP RQ, Thermofisher Scientific, USA).
Data analysis	All statistical analyses were performed on Graphpad Prism (version 8.0) or Excel 2016; The 1H NMR data were analyzed on MestReNova (version 6.1.1); All flow cytometry data were analyzed on FlowJo software (version 10.0.7); Molecular Imaging Software (version 2.0, Bruker) was used to analyse living fluorescent images; ZEN2 (blueedition) software (Version 2.1), NIS-Elements C acquisition analysis software, Cellsens software (Version 2.2) and ImageJ (version 1.48) were used to analyze confocal images.

For manuscripts utilizing custom algorithms or software that are central to the research but not yet described in published literature, software must be made available to editors and reviewers. We strongly encourage code deposition in a community repository (e.g. GitHub). See the Nature Portfolio guidelines for submitting code & software for further information.

Data

Policy information about availability of data

All manuscripts must include a data availability statement. This statement should provide the following information, where applicable:

- Accession codes, unique identifiers, or web links for publicly available datasets
- A description of any restrictions on data availability
- For clinical datasets or third party data, please ensure that the statement adheres to our policy

The raw data can be found in the Source Data. Source data are provided with this paper. The authors declare that all data supporting the findings of this study are available within the paper, Supplementary information. The uncropped versions of immunoblot images has been provided in Source Data file.

Field-specific reporting

Please select the one below that is the best fit for your research. If you are not sure, read the appropriate sections before making your selection.

☒ Life sciences ☐ Behavioural & social sciences ☐ Ecological, evolutionary & environmental sciences

For a reference copy of the document with all sections, see nature.com/documents/nr-reporting-summary-flat.pdf

Life sciences study design

All studies must disclose on these points even when the disclosure is negative.

Sample size	All in vitro data were done at least in triplicate and SD were reported as stated in the figure and figure legends. For in vivo experiments, 3 to 10 mice were sufficient to identify differences between groups with at least 80% power and a 5% significance level. Sample size was estimated on the basis of similar research reported in the literature (references below). Details regarding sample size of all experiments are provided in the Methods section and figure legends.<br>1. H. Li, et al. Disrupting tumour vasculature and recruitment of aPDL1-loaded platelets control tumour metastasis. Nat Commun. 2021, 12, 2773.<br>2. Q. Chen, et al. In situ sprayed bioresponsive immunotherapeutic gel for post-surgical cancer treatment. Nat Nanotechnol. 2019, 14, 89-97.
Data exclusions	No data were excluded from the analysis.
Replication	Experiments were replicated and experimental findings were reproducible. Experimental repeat numbers are reported in Figure Legends.
Randomization	All samples, cells and mice were allocated randomly into experimental groups.
Blinding	No formal blinding was used. The investigator organizing the experimental groups and involved in sample collection was not blinded; however, colleagues aiding in data collection were blinded. Most of the studies contained multiple steps (including the material preparation, tumor therapeutics, and so on) and the scientists must keep careful track of every condition. It would be exceedingly difficult to blind such studies.

Reporting for specific materials, systems and methods

We require information from authors about some types of materials, experimental systems and methods used in many studies. Here, indicate whether each material, system or method listed is relevant to your study. If you are not sure if a list item applies to your research, read the appropriate section before selecting a response.

Materials & experimental systems

n/a	Involved in the study	
☐	☒	Antibodies
☐	☒	Eukaryotic cell lines
☒	☐	Palaeontology and archaeology
☐	☒	Animals and other organisms
☒	☐	Human research participants
☒	☐	Clinical data
☒	☐	Dual use research of concern

Methods

n/a	Involved in the study	
☒	☐	ChIP-seq
☐	☒	Flow cytometry
☒	☐	MRI-based neuroimaging

Antibodies

Antibodies used	The following primary antibodies were used for flow cytometry. They are listed as antigen first, following by supplier, catalog number, clone/lot number and dilution ratio for staining as applicable:<br>1. Purified anti-mouse CD16/32 Antibody, Biolegend, cat. no. 101302, Clone: 93, (1:100 dilution);

2. Brilliant Violet 510™ anti-mouse CD45 Antibody, Biolegend, cat. no. 103137, Clone: 30-F11, (1:100 dilution);
3. Brilliant Violet 711™ anti-mouse CD8a Antibody, Biolegend, cat. no. 100748, Clone: 53-6.7, (1:100 dilution);
4. APC anti-mouse CD3 Antibody, Biolegend, cat. no. 100236, Clone: 17A2, (1:150 dilution);
5. Brilliant Violet 421™ anti-mouse FOXP3 Antibody, Biolegend, cat. no. 126419, Clone: MF-14, (1:100 dilution);
6. PE anti-mouse CD206 Antibody, Biolegend, cat. no. 141706, Clone: C068C2, (1:100 dilution);
7. Brilliant Violet 605™ anti-mouse CD11b Antibody, Biolegend, cat. no. 101237, Clone: M1/70, (1:100 dilution);
8. PE/Cyanine7 anti-mouse F4/80 Recombinant Antibody, Biolegend, cat. no. 157308, Clone: QA17A29, (1:100 dilution);
9. Brilliant Violet 650™ anti-mouse CD25 Antibody, Biolegend, cat. no. 102037, Clone: PC61, (1:100 dilution);
10. PerCP/Cyanine5.5 anti-mouse Ly-6G/Ly-6C (Gr-1) Antibody, Biolegend, cat. no. 108428, Clone: RB6-8C5, (1:100 dilution);
11. APC/Cyanine7 anti-mouse CD86 Antibody, Biolegend, cat. no. 105029, Clone: GL-1, (1:100 dilution);
12. FITC anti-mouse CD80 Antibody, Biolegend, cat. no. 104705, Clone: 16-10A1, (1:100 dilution);
13. Alexa Fluor® 700 anti-mouse I-A/I-E (MHC-II) Antibody, Biolegend, cat. no. 107622, Clone: M5/114.15.2, (1:300 dilution);
14. PE/Cyanine5 anti-mouse CD19 Antibody, Biolegend, cat. no. 115510, Clone: 6D5, (1:150 dilution);
15. BUV563 anti-mouse CD4 Antibody, BD Bioscience, cat. no. 741217, Clone: RM4-5, (1:200 dilution).

The following primary antibodies were used for Western Blot and Immunohistochemical staining. They are listed as antigen first, following by supplier, catalog number, clone/lot number and dilution ratio for staining as applicable.
1. Anti-Ki67 antibody, Abcam, cat. no. ab16667, Clone: Rabbit monoclonal [SP6], (1:200 dilution);
2. Anti-GAPDH antibody, Abcam, cat. no. ab181602, Clone: rabbit monoclonal [EPR16891], (1:10000 dilution);
3. Anti-MMP3 antibody, Abcam, cat. no. ab52915, Clone: rabbit monoclonal [EP1186Y], (1:1000 dilution);
4. Anti-MMP9 antibody, Abcam, cat. no. ab228402, Clone: rabbit monoclonal [EPR22140-154], (1:1000 dilution);
5. Anti-MMP2 antibody, Abcam, cat. no. ab86607, Clone: mouse monoclonal [6E3F8], (1:1000 dilution);
6. Goat anti-rabbit IgG H&L (HRP), Abcam, cat. no. ab6721, (1:2000 dilution);
7. Goat anti-mouse IgG H&L (HRP), Abcam, cat. no. ab6789, (1:2000 dilution);
8. Anti-iNOS antibody, Abcam, cat. no. ab49999, Clone: mouse monoclonal [NOS-IN], (1:1000 dilution);
9. Anti-Arg antibody, Abcam, cat. no. ab96183, Clone: rabbit monoclonal antibody, (1:500 dilution);
10. Goat anti-mouse IgG H&L (Alexa Fluor® 647), Abcam, cat. no. ab150115, Clone: goat monoclonal antibody, (1:1000 dilution);
11. Goat anti-rabbit IgG H&L (Alexa Fluor® 488), Abcam, cat. no. ab150077, Clone: goat monoclonal antibody (1:500 dilution);
12. FITC anti-mouse CD8a Antibody, Biolegend, cat. no. 100706, Clone: 53-6.7 (1:500 dilution);
13. PE/DazzleTM 594 anti-mouse CD4 Antibody, Biolegend, cat no. 100456, clone: GK1.5, (1:500 dilution);
14. APC anti-mouse CD86 Antibody, Biolegend, cat no. 105012, clone: GL-1, (1:500 dilution);
15. PE anti-mouse CD206 Antibody, Biolegend, cat no. 141706, clone: C068C2, (1:500 dilution).

Validation

All antibodies were verified by the supplier and each lot has been quality tested. All validation statements can be found on the respective antibody website:
1. Purified anti-mouse CD16/32 Antibody: https://www.biolegend.com/en-us/search-results/purified-anti-mouse-cd16-32-antibody-190;
2. Brilliant Violet 510™ anti-mouse CD45 Antibody: https://www.biolegend.com/en-us/products/brilliant-violet-510-anti-mousecd45-antibody-7995;
3. Brilliant Violet 711™ anti-mouse CD8a Antibody: https://www.biolegend.com/en-us/products/brilliant-violet-711-anti-mousecd8a-antibody-7926;
4. APC anti-mouse CD3 Antibody: https://www.biolegend.com/en-us/products/apc-anti-mouse-cd3-antibody-8055;
5. Brilliant Violet 421™ anti-mouse FOXP3 Antibody: https://www.biolegend.com/en-us/products/brilliant-violet-421-antimouse-foxp3-antibody-12143;
6. PE anti-mouse CD206 Antibody: https://www.biolegend.com/en-us/products/pe-anti-mouse-cd206-mmr-antibody-7424;
7. Brilliant Violet 605™ anti-mouse CD11b Antibody: https://www.biolegend.com/en-us/products/brilliant-violet-605-antimouse-human-cd11b-antibody-7637;
8. PE/Cyanine7 anti-mouse F4/80 Recombinant Antibody: https://www.biolegend.com/en-us/products/pecyanine7-anti-mousef480-recombinant-antibody-18757;
9. Brilliant Violet 650™ anti-mouse CD25 Antibody: https://www.biolegend.com/en-us/products/brilliant-violet-650-anti-mousecd25-antibody-7640;
10. PerCP/Cyanine5.5 anti-mouse Ly-6G/Ly-6C (Gr-1) Antibody: https://www.biolegend.com/en-us/products/percp-cyanine5-5-anti-mouse-ly-6g-ly-6c-gr-1-antibody-4286;
11. APC/Cyanine7 anti-mouse CD86 Antibody: https://www.biolegend.com/en-us/products/apc-cyanine7-anti-mouse-cd86-antibody-6554;
12. FITC anti-mouse CD80 Antibody: https://www.biolegend.com/en-us/products/fitc-anti-mouse-cd80-antibody-41;
13. Alexa Fluor® 700 anti-mouse I-A/I-E (MHC-II) Antibody: https://www.biolegend.com/en-us/products/alexa-fluor-700-antimouse-i-a-i-e-antibody-3413;
14. PE/Cyanine5 anti-mouse CD19 Antibody: https://www.biolegend.com/en-us/products/pe-cyanine5-anti-mouse-cd19-antibody-1531;
15. BUV563 anti-mouse CD4 Antibody: https://www.bdbiosciences.com/en-us/products/reagents/flow-cytometry-reagents/research-reagents/single-color-antibodies-ruo/buv563-rat-anti-mouse-cd4.741217;
16. Anti-Ki67 antibody: https://www.abcam.cn/ki67-antibody-sp6-ab16667.html;
17. Anti-GAPDH antibody: https://www.abcam.cn/gapdh-antibody-epr16891-loading-control-ab181602.html;
18. Anti-MMP3 antibody: https://www.abcam.cn/mmp3-antibody-ep1186y-ab52915.html;
19. Anti-MMP9 antibody: https://www.abcam.cn/mmp9-antibody-epr22140-154-ab228402.html;
20. Anti-MMP2 antibody: https://www.abcam.cn/mmp2-antibody-6e3f8-ab86607.html;
21. Goat anti-rabbit IgG H&L (HRP): https://www.abcam.cn/goat-rabbit-igg-hl-hrp-ab6721.html;
22. Goat anti-mouse IgG H&L (HRP): https://www.abcam.cn/goat-mouse-igg-hl-hrp-ab6789.html;
23. Anti-iNOS antibody: https://www.abcam.cn/inos-antibody-nos-in-ab49999.html;
24. Anti-Arg antibody: https://www.abcam.cn/liver-arginase-antibody-ab96183.html;
25. Goat anti-mouse IgG H&L (Alexa Fluor® 647): https://www.abcam.cn/goat-mouse-igg-hl-alexa-fluor-647-ab150115.html;
26. Goat anti-rabbit IgG H&L (Alexa Fluor® 488): https://www.abcam.cn/goat-rabbit-igg-hl-alexa-fluor-488-ab150077.html;
27. FITC anti-mouse CD8a antibody: https://www.biolegend.com/en-us/products/fitc-anti-mouse-cd8a-antibody-153;
28. PE/DazzleTM 594 anti-mouse CD4 antibody: https://www.biolegend.com/en-us/products/pe-dazzle-594-anti-mouse-cd4-antibody-11949;

29. APC anti-mouse CD86 antibody: https://www.biolegend.com/en-us/products/apc-anti-mouse-cd86-antibody-2896;
30. PE anti-mouse CD206 antibody: https://www.biolegend.com/en-us/products/pe-anti-mouse-cd206-mmr-antibody-7424.

Eukaryotic cell lines

Policy information about cell lines

Cell line source(s)	4T1 murine mammary carcinoma cells was obtained from American Type Culture Collection (ATCC). 4T1-GFP were constructed by infecting GFP-encoding lentivirus into 4T1 cells and then selected by puromycin (2 ug/mL).
Authentication	Authentification was performed by ATCC for 4T1 cell line (Method: STR profiling).
Mycoplasma contamination	All cell lines were tested for mycoplasma contamination. No mycoplasma contamination was found.
Commonly misidentified lines (See ICLAC register)	No commonly misidentified cell lines were used in the study.

Animals and other organisms

Policy information about studies involving animals; ARRIVE guidelines recommended for reporting animal research

Laboratory animals	Female BALB/c mice (6-8 weeks old) were purchased from Vital River Laboratory Animal Technology Co., Ltd. (Beijing, China). Animals were housed at approximately 22 ± 2 degrees centigrade, humidity 50±10% on a 12 h light/ 12 h dark cycle.
Wild animals	The study did not involve wild animals.
Field-collected samples	The study did not involve samples collected from the field.
Ethics oversight	All the animal experiments were approved by the Animal Care and Use Committee at South China University of Technology (SCUT), and every effort was made to minimize suffering from experiments.

Note that full information on the approval of the study protocol must also be provided in the manuscript.

Flow Cytometry

Plots

Confirm that:

☒ The axis labels state the marker and fluorochrome used (e.g. CD4-FITC).

☒ The axis scales are clearly visible. Include numbers along axes only for bottom left plot of group (a 'group' is an analysis of identical markers).

☒ All plots are contour plots with outliers or pseudocolor plots.

☒ A numerical value for number of cells or percentage (with statistics) is provided.

Methodology

Sample preparation	Tumor tissues were harvested, minced and incubated with RPMI-1640 medium containing 10% FBS (v/v), collagenase type I (1 mg/mL), hyaluronidase (100 μg/mL) and DNase I (100 μg/mL) at 37 ℃ for 25 min with persistent agitation. Digested cells were passed through a 40-μm nylon mesh and collected by centrifugation at 1,500 revolutions per minute for 10 min, followed by Red Blood Cell (RBC) lysis. 100 μL of cell suspension (2.0 × 107 cells/mL) was used for flow cytometry detection.
Instrument	BD LSRFortessa™ flow cytometer
Software	FlowJo software v10.0.7.
Cell population abundance	No sorting was performed.
Gating strategy	The detailed gating strategy could be found in the supplementary Fig. 26. Immune cell population was gated based on the expression of CD45 (118138 events in gate), CD45+ cells were further gated to determine F4/80-Gr-1- lymphocyte cells (13734 events in gate), and F4/80+Gr-1- macrophage cells (33703 events in gate). The F4/80-Gr-1- lymphocyte cells were further gated to determine CD3+ T cells (6697 events in gate), CD3+CD8+ (2358 events in gate), CD3+CD4+ T cells (4005 events in gate) and CD3+CD4+Foxp3+ Treg cells (2568 events in gate). The F4/80+Gr-1- cells were further gated to determine CD11b+CD86+MHC-II+ M1 macrophages (3157 events in gate) and CD11b+CD206+ M2 macrophages (5768 events in gate). Isotype controls were used for identifying the non-specific background.

☒ Tick this box to confirm that a figure exemplifying the gating strategy is provided in the Supplementary Information.
